# Evidence use in decision-making on introducing innovations: a systematic scoping review with stakeholder feedback

**DOI:** 10.1186/s13012-017-0669-6

**Published:** 2017-12-04

**Authors:** Simon Turner, Danielle D’Lima, Emma Hudson, Stephen Morris, Jessica Sheringham, Nick Swart, Naomi J. Fulop

**Affiliations:** 1 Centre for Primary Care, Division of Population Health, Health Services Research and Primary Care, School of Health Sciences, Faculty of Biology, Medicine and Health, University of Manchester, Manchester, UK; 2 Department of Clinical, Educational and Health Psychology, UCL Centre for Behaviour Change, University College London, 1-19 Torrington Place, London, UK; 3 Department of Applied Health Research, University College London, 1-19 Torrington Place, London, UK

**Keywords:** Evidence, Innovation, Service improvement, Decision-making, Qualitative, Professions, Organisations, Local systems

## Abstract

**Background:**

A range of evidence informs decision-making on innovation in health care, including formal research findings, local data and professional opinion. However, cultural and organisational factors often prevent the translation of evidence for innovations into practice. In addition to the characteristics of evidence, it is known that processes at the individual level influence its impact on decision-making. Less is known about the ways in which processes at the professional, organisational and local system level shape evidence use and its role in decisions to adopt innovations.

**Methods:**

A systematic scoping review was used to review the health literature on innovations within acute and primary care and map processes at the professional, organisational and local system levels which influence how evidence informs decision-making on innovation. Stakeholder feedback on the themes identified was collected via focus groups to test and develop the findings.

**Results:**

Following database and manual searches, 31 studies reporting primary qualitative data met the inclusion criteria: 24 were of sufficient methodological quality to be included in the thematic analysis. Evidence use in decision-making on innovation is influenced by multi-level processes (professional, organisational, local system) and interactions across these levels. Preferences for evidence vary by professional group and health service setting. Organisations can shape professional behaviour by requiring particular forms of evidence to inform decision-making. Pan-regional organisations shape innovation decision-making at lower levels. Political processes at all levels shape the selection and use of evidence in decision-making.

**Conclusions:**

The synthesis of results from primary qualitative studies found that evidence use in decision-making on innovation is influenced by processes at multiple levels. Interactions between different levels shape evidence use in decision-making (e.g. professional groups and organisations can use local systems to validate evidence and legitimise innovations, while local systems can tailor or frame evidence to influence activity at lower levels). Organisational leaders need to consider whether the environment in which decisions are made values diverse evidence and stakeholder perspectives. Further qualitative research on decision-making practices that highlights how and why different types of evidence come to count during decisions, and tracks the political aspects of decisions about innovation, is needed.

**Electronic supplementary material:**

The online version of this article (10.1186/s13012-017-0669-6) contains supplementary material, which is available to authorized users.

## Background

A range of evidence informs decision-making on innovation in health care, including formal research findings [1], local data [2] and professional experience [3]. However, cultural and organisational factors often prevent the translation of evidence for innovations into practice [4]. The health care decision-making and innovation studies literature has shown that the role of evidence in decision-making on innovation is influenced by the characteristics of evidence, e.g. accessibility of economic evaluation [5], and processes at the individual level. Individual level processes include preferences for evidence [6], how its interpreted [7–9], and individuals’ credibility, personality and experience when sharing evidence [10–12]. The role of processes at the wider professional group (e.g. preferences, professional interests and power dynamics) and organisational level has been reviewed with regard to the diffusion of innovations [13, 14], but not in relation to their impact on how evidence informs adoption decisions specifically. In diffusion of innovations theory, decision-making is said to pass through five stages in relation to innovations [15]. In relation to the scope of this review, evidence is relevant at the stages of ‘knowledge’ (information sought about the innovation), ‘persuasion’ (information sought to reduce uncertainty, e.g. scientific evaluations, peers’ opinions) and ‘decision’ (evidence of trialling of new idea). While diffusion of innovations theory highlights that a variety of evidence influences adoption decisions, it does so predominantly in relation to the individual’s attitude toward an innovation to the neglect of decision-making processes at wider contextual levels [16]. There is no consensus about the ways in which processes at the professional group [6, 17–19], organisational [20] and local system level [21], influence the use of evidence in decisions to adopt innovations.

The purpose of this systematic scoping review was to understand how processes at different levels influence the use of evidence in decision-making on health care innovations by (1) mapping processes at the professional, organisational and local system levels which influence how evidence informs decision-making on innovation and (2) collecting stakeholder feedback to validate and develop the findings. The review focussed on primary qualitative studies as these were appropriate for understanding how and why contextual processes at different levels influence evidence use in decision-making. Qualitative studies can capture this context by focusing on processes and experiences of innovation at the professional group, organisational (defined as an organisation’s decision-making systems, culture and management practices) and local system level (the embedding of organisations in the wider environment or context) [22].

## Methods

Literature on evidence use in decision-making on innovation was identified, selected and analysed using a scoping review approach [23–25], which involved six stages: (1) identifying the research question, (2) defining the scope of the review, (3) study selection, (4) charting the data, (5) reporting the results and (6) stakeholder consultation. We used recommendations for undertaking each stage systematically [24], including using two researchers to independently review articles for inclusion and defining the consultation stage’s purpose and types of stakeholder to involve. The review was completed in accordance with Preferred Reporting Items for Systematic Reviews and Meta-Analyses (PRISMA) (Additional file 1). A review protocol was not registered. The six stages used in this review are described below.

### Stage 1: identifying the research question

This review’s guiding research question was ‘How do decision-making processes at the professional group, organisational, and local system level influence the use of evidence in decisions to adopt innovations in acute and primary health care?’ Selection of these three levels reflects the theorised influence of these aspects of the local context during quality improvement processes [16, 26], with our specific research question seeking to understand their influence on evidence use in decision-making on innovation. In addressing this question, we defined the terms ‘evidence’, ‘innovation’ and ‘decision-making’ and how they would be captured in the review.

#### Evidence

The conceptual literature on evidence use highlights that a range of evidence may impact on decisions about innovation or improvement. The evidence-based medicine (EBM) movement, and its extension into other areas, including health care management, has been influential in how evidence is conceptualised. EBM involves providing care by integrating individual clinical expertise, evidence from systematic research and patient choice [27]. Those critical of EBM suggests that alternative forms of evidence, such as patients’ views on innovations [28], and qualitative research that provides insight into real-world contexts and participants’ interpretations [29], should be recognised for their role in decision-making. We adopted an inclusive and broad working definition of evidence that included diverse forms of information, including academic research findings, patient experience, professional opinion, clinical guidance and local data.

#### Innovation

Innovation was defined broadly as the development and implementation of new ideas, products, processes or organisational forms [30, 31]. Our use of the term in relation to health care encompasses service or quality improvement. No claim was made a priori about innovation efficacy or effectiveness, as this was assumed to vary by innovation and may not have been assessed. Although the term ‘innovation’ may not be used in everyday practice to describe changes to product, process or organisational form, these were still considered as potential forms of innovation. These include product innovations such as robotic surgery, process innovations including hospital-wide patient safety programmes and new organisational forms, e.g. reconfiguration of acute stroke services. Innovations might relate to service provision or commissioning and be introduced at a system-wide level or be locally led. Studies of innovations that were not discussed in relation to their adoption within a service or delivery context were excluded, e.g. early phase development of new drugs or medical devices. Conversely, a study of pharmaceutical innovation we included examined decision-making on adopting new drugs for use in clinical practice [8].

#### Decision-making

This review included decisions about whether to adopt new innovations or spread existing innovations up to the point of implementation (implementation itself was considered relevant where it influenced adoption decisions). We adopted a ‘processual’ approach to the study of decision-making on innovation, understanding it as an ongoing, often non-linear process that unfolds over time [32]. Different approaches to decision-making are possible which may influence how evidence is used, ranging from more authoritarian to participatory [15, 33]. We focussed on decision-making at the micro (professional) and meso (organisational/local system) levels.

### Stage 2: defining the scope of the review

The scoping review aimed to identify examples of evidence use in decisions about innovation (or related improvement activity) from studies conducted in relation to the UK National Health Service (NHS) and health systems internationally. The review’s focus was on the influence of interactions between evidence use and processes at the micro (professional) and meso (organisational/system) level on decisions to introduce or diffuse innovations. Selection criteria were defined a priori and applied by two researchers to the title/abstract, and then full text, of potentially relevant papers. The review focused on decision-making on innovations in health care services (acute, primary) and multi-sectoral studies including health care services. Studies that examined decisions about innovation or other improvement activities, but did not refer to evidence use, were excluded. Only studies conducted in Organisation for Economic Co-operation and Development (OECD) countries were included to aid comparability of health care systems. Only English language references, published since 2006, were included. This date was chosen because it coincided with recognition among policymakers and researchers of the challenges of mobilising evidence in health care, including concerns about traditional models of translating research into practice [4] and critical perspectives on EBM [34]. Studies of decision-making at the national (macro) health system level and public health or prevention were excluded as reviews exist in these areas [35–37]. This review focussed on decision-making on innovation by professional groups and organisations within local health systems, rather than the related field of policy development, including intervention design, at the national health system level [38]. An online bibliographic database (EPPI-reviewer 4) was used to store and manage references [39].

### Stage 3: study selection

To identify relevant literature, social science and biomedical databases were searched in May 2016. A search strategy was created for MEDLINE. Search terms in the title or abstract were ‘innovation or improvement’, ‘decision or decision-making’, ‘evidence’ and ‘health care’. Medical Subject Headings (MeSH) were also used, which included ‘diffusion of innovation’, ‘translational medical research’, ‘Evidence-based practice’, ‘knowledge bases’ and ‘decision making, organizational’. The search was adapted for other databases: Embase, PsycINFO, Scopus, Health Management Information Consortium (HMIC), and EBSCO Business Source Complete (see Additional file 2). Suggestions of relevant literature were made by the wider study’s project advisory group (PAG) [40], which included academics with relevant expertise, practitioners with clinical insight on delivering service change and patient representatives.

### Stage 4: charting the data

A data extraction framework was used to chart information from the included studies, including setting, type of innovation, characteristics of evidence and quality assessment (Additional file 3); study type and methods, aim and objectives, and professional, organisational and local system processes that influenced evidence use (Additional file 4).

### Stage 5: reporting the results

The review combined aggregative/integrative and configurative/interpretative approaches to the synthesis of evidence [41–43]. First, thematic analysis by two researchers was used to summarise findings from existing studies (aggregative) by tabulating data extracted from the qualitative studies. Analysis focused on the types of evidence referred to multi-level influences on evidence use and sector/stakeholder perspective. Second, using meta-case analysis of the compiled literature, new ideas and themes, i.e. novel third order concepts [44], were developed during the review (configurative). The concept of interactions between levels (professional, organisational, local system), and their influence on evidence use, emerged from the meta-case analysis in which relationships between the tabulated themes were explored.

### Stage 6: stakeholder consultation

To test and develop the results from the scoping review, four focus groups, with 18 participants in total, were organised with mixed stakeholder groups comprising acute care providers (4), primary care providers (3), service commissioners (3), patient representatives (5) and knowledge intermediaries (3). Reporting of the focus groups (Additional file 5) was informed by consolidated criteria for reporting qualitative research (COREQ) checklist [45].

## Results

**Fig. 1:**
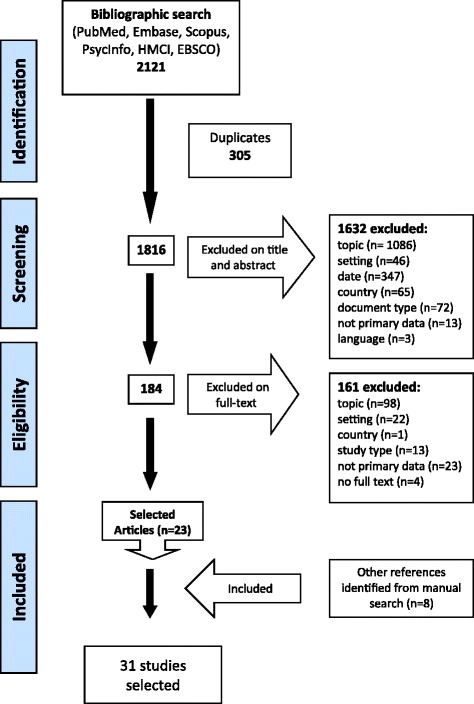
PRISMA flow diagram

The database search produced 1816 results, after duplicates were excluded. After screening by title and abstract using the inclusion and exclusion criteria, 184 articles were identified for full-text screening, 23 of which were selected for data extraction (Fig. 1). A manual search for relevant studies conducted after the database search, based on searching key journals (*Social Science & Medicine*, *BMJ Quality & Safety*, *Implementation Science*, *Sociology of Health & Illness*) and suggestions by PAG members, including book chapters, identified eight additional studies for inclusion, meaning 31 studies were reviewed.

The quality of studies was assessed using the Critical Appraisal Skills Programme (CASP) Qualitative Checklist [46], which includes nine questions for assessing the validity of study findings numerically and a tenth, non-quantifiable question for judging the overall relevance or value of the research (recognising that the checklist represents a series of inter-related questions for assessing study quality). After reviewing all of the studies using the CASP checklist, we agreed that seven studies should be considered lower quality studies. This assessment took into account how each study performed against the numerical questions and making a value judgement about the quality of each study as a whole (question ten). Those seven studies were excluded from the thematic analysis due to low confidence in the validity of results (studies shown in ‘greyed out’ rows in the data extraction tables).

### Study characteristics

A summary of the studies’ characteristics is provided in Additional file 3. The majority of the studies was conducted in the UK (14), followed by Canada (5), Australia (5), the USA (3), Sweden (1) and Italy (2). An interview-based study [47], of lower methodological quality, included participants from Australia, Denmark, Ireland, the Netherlands, Slovenia, Switzerland, Spain and Sweden. The types of innovation examined were technological innovation (6), staff and patient involvement in quality improvement (4), responses to clinical guidelines or tools (7), organisational innovation including quality improvement programmes (6) and technology assessment and priority setting (8). The studies covered acute care (16), primary care (11), commissioning (8), health and social care (2) and mental health (1). Nearly all (28) of the 31 studies employed qualitative interviews. In combination with interviews, these studies used observations (9), documentary analysis (9), focus groups (4) and surveys (5). Of the remaining three studies, two relied on observations and one did not specify data collection methods within a case study approach. Research evidence was the most cited form of evidence in decision-making on innovation (19 studies); other forms of evidence were professional experience (15), local data (12), national guidance (7), translational information, e.g. education/ summaries (4), patient involvement (3) and expert opinion (3).

There were 24 studies of sufficient quality to be included in the thematic analysis. Thematic analysis examined how processes at different levels (professional, organisational, local system) influenced the use of evidence in decision-making on innovation (Additional file 4).

### Professional level processes influencing evidence use

#### Preferences for evidence

Preferences for evidence varied by professional group and across health care sectors. Service payers (commissioners) drew on a range of evidence, including alternative evidence such as patient stories, and prioritised local need for innovations over research evidence [14, 48]. In the acute sector, nurses tended to combine practical (‘how to’) and scientific (‘principles’) knowledge, while medical professionals placed greater weight on the latter [49]. In primary care, general practitioners (GPs) did not necessarily privilege scientific evidence; research-based studies were contested by GPs as results were weighed up against their knowledge of patient need [50]. Evidence can be given different meanings by different stakeholders resulting in uncertainty about whether evidence was lacking, was not of good quality, or was limited [51].

#### Professional interests

Some studies highlighted that decisions to develop and adopt innovations reflected professional interests. A study of surgical innovation found that surgeons ‘spoke for’ patients by introducing new techniques that would ‘make sense’ for them, even though supporting data were lacking [52]. A study of remote care (telecare) found that evidence was actively constructed and adapted to fit managers’ agendas [53]. There was recognition that evidence could be ‘gamed’ whereby evidence was found to support decisions that had already been taken [54]. Professional interests could influence how different stakeholders responded to evidence. A primary care study of the failure to implement externally mandated rules, National Service Frameworks, was linked by GPs to concerns about the accessibility of evidence (e.g. document length, complexity, local applicability), but the authors suggested these were mere ‘constructions’ because the frameworks did not fit in with GPs’ professional identities [55].

#### Power dynamics

Power dynamics between different professional groups influenced evidence use. A study of interventions to improve prescribing practice in primary care found that managers leading the programme privileged scientific evidence, while attempting to marginalise GPs’ clinical and experiential knowledge [50]. Similarly, managers used evidence to decline clinicians’ ‘unreasonable’ requests for innovation in the area of robotic surgery [56]. Conversely, a study of committees considering technology coverage found that clinicians, especially those with powerful personalities, were able to influence the committees [8]. Even where decision-makers agreed on the evidence base for an intervention, there could be disagreement based on practitioner and patient judgements about how such evidence should be used to make decisions and/or change services [57].

The stakeholder feedback indicated that professional processes influenced decision-making. They confirmed that professional credibility of those presenting evidence, as well as clinical leadership and ‘soft’ persuasion skills and relationship-building (including ‘endless discussions’), encouraged evidence to be taken seriously and acted upon. There was recognition that preferences for evidence varied by stakeholder and therefore the same evidence often need to be framed differently to influence different stakeholders, particularly the needs of commissioners or funders of potential innovations, ‘as everybody has different buttons’. The ongoing process of building relationships during decision-making was more apparent in the stories of innovation shared in the focus groups than in the literature review (due perhaps to a lack of processual studies in extant literature) [6].

### Organisational level processes influencing evidence use

#### Organisational roles

Organisations contributed to assessing non-clinical aspects of innovation. Along with evidence of clinical need or effectiveness, budgetary and financial issues were important in decision-making [17, 49]. Organisations enabled stakeholder involvement in decision-making, including staff [11], which aided subsequent implementation [10]. Stakeholder involvement in quality improvement projects, particularly patients and the public, was supported by effective communication channels and a ‘non-hierarchical’ environment for decision-making [12]. Centralised approaches to decision-making, coupled with a lack of communication, inhibited evidence use by planners within regional health authorities in Canada [54]. Organisations limited innovations proposed by clinicians and other stakeholders where evidence was lacking: funding for surgical innovation was cut by a hospital due to a lack of evidence on cost, safety and effectiveness, despite local surgeons’ perceptions that it improved patient outcomes and safety [52].

#### Organisational facilitators

A number of organisational facilitators to evidence use in decisions about innovation were identified. In a study of technology adoption within hospitals, access to and use of research evidence in decision-making was enabled by organisational processes, including infrastructure redevelopment projects and an emphasis on collaboration [6]. In a study of priority setting within a provincial health services authority, evidence use was enabled by strong leadership, a culture of openness and learning, and commitment to being ‘data-driven’ [9]. The importance of research culture was borne out by a study of a multi-systemic therapy, where entrepreneurial leaders of adopter sites suggested that they could make decisions to adopt innovations more readily than non-adopters because they were more aware of the evidence base [58]. Innovation was supported by creating leadership for change, which included marketing evidence of benefit and building a supportive community of practice [59]. Another study highlighted the importance of involving both managers and clinicians in decision-making [60]. The chairs of decision-making committees moderated the use of evidence type. A study of networks responsible for enhancing multidisciplinary cancer care found that some chairs steered the conversation more to scientific and technical themes at the expense of narrative perspectives [61].

#### Organisational barriers

Underlying organisational issues could act as barriers to introducing innovations [55]. A lack of time, resources and pressures inhibited evidence use [54]. A lack of authority to make changes to processes also influenced decision-making [9]. In some contexts, organisations were not receptive to change. A study of telehealth services found that its spread was limited in two out of five cases by a lack of alignment between the adopting organisations’ values and managers’ agendas [53]. One study suggested that those proposing innovations should ensure these were aligned with other activities already familiar to decision-making stakeholders [57]. Another study found that involvement processes for enabling patient organisations to participate in funding decisions were inadequate for including patients’ experiences [62].

#### Organisational politics

Organisational politics influenced the type of evidence accessed and how it was interpreted. The use of economic evaluation by committees making technology coverage decisions was limited by unclear relationships with resource allocators, an explicitly political decision-making process, and poorly specified decision-making criteria [8]. A study of commissioners’ information use [48] found that organisational processes changed the original information gathered during decision-making (evidence was re-framed over time to suit competing agendas).

The stakeholder feedback confirmed that an innovation was more likely to be adopted when it was aligned with organisational needs, e.g. when it is a priority (including meeting external targets or initiatives) and it addressed a clear, practical problem. The focus groups elaborated on the influence of the decision-making approach taken in relation to innovations of different scales. There was recognition that large-scale change was difficult because a wide range of stakeholders were often involved and that evidence often showed both pros and cons. The stakeholders discussed different approaches to organisational decision-making; ‘autocratic’ as opposed to ‘democratic’ organisations were better placed to introduce change, but once a decision had been made, there was the challenge of getting a change accepted and having a culture that valued evidence was deemed important for this.

### Local system level processes influencing evidence use

#### External pressures

External pressures, including system restructuring [57], meeting policy targets [6] and budgetary constraint [7, 17, 59], influenced how evidence was used in decisions about innovation. The political context influenced decision-making [9], e.g. decisions needed to stand up to external scrutiny [48]. Such pressures could lead to an emphasis on ‘what works’ in making adoption decisions over use of rigorous evidence [6]. One study reported staff being overwhelmed when using evidence to make decisions about changing services due to competing priorities and variable managerial support during major external change [57]. A context of austerity could encourage evidence to be viewed differently. To receive funding, home telehealth services needed to demonstrate savings or efficiencies as well as evidence of benefit [59]. Due to the need to consider rationing of the health care system, another study argued that decision-makers viewed economic evaluation narrowly–based on budgetary impact and costs rather than cost effectiveness [7]. Another study found that financial and resource issues facing commissioners made them more conservative when changing services in response to new national guidelines [60].

#### Pan-regional organisations

Pan-regional organisations influenced how evidence was used in decisions about innovation. On the one hand, such organisations had a downward influence on evidence use in local decision-making. A study of a collective primary care organisation showed how it influenced GPs’ prescribing practice by emphasising evidence that appealed to this professional group (i.e. improving quality through prescribing targets), while deemphasizing the contribution of the interventions to cost containment which appealed less to GPs [50]. A national improvement programme was the source of evidence for improving ward productivity, which had a national organisational profile and established links with providers, aiding adoption [63]. However, a regional health technology advisory group in Sweden had less influence on decision-making because it was not embedded sufficiently in local decision-making [51]. On the other hand, an upward relationship from the organisational to local system level existed whereby pan-regional organisations helped legitimise local innovations or encourage disinvestment. Hospitals’ participation in a national improvement campaign afforded external validation of decision-makers’ opinions at a local level, aiding programme commitment [56]. One Canadian study found that a regional body was used by a hospital to justify withdrawing funding for an innovation, based on a perceived lack of evidence [52].

#### Widening stakeholder involvement

Participation in external systems or networks enabled a wider range of potential stakeholders to inform decision-making on innovation. However, taking into account a range of external stakeholders’ views could hinder implementing innovations based on formal evidence alone; the politics of decision-making could be more important than evidence, including the assessment of likely public perceptions of decisions taken [53]. Decision-making could be enhanced through the use of deliberative involvement processes enabling multiple stakeholders to participate [62].

The stakeholder feedback indicated that organisations at the local system level played an important role in enabling innovation. The backing of research organisations and other knowledge intermediaries, e.g. Academic Health Science Networks and CLAHRCs, provided a facilitating role–one participant referred to them as ‘ambassadors’ for innovation–that could help to bring together relevant stakeholders. The role of intermediaries in mobilising evidence for innovations by brokering social relationships came through more clearly in the focus groups than in the literature review, possibly because studies of knowledge mobilisation tend to consider implementation processes (which were excluded from the review) rather than adoption decisions [21]. The focus groups confirmed the importance of the political context, especially perceived pressure to reduce or control costs, and the need for evidence for innovations to align with those setting the political direction.

## Discussion

### Summary

This is the first systematic scoping review to examine how processes at multiple levels (professional, organisational, local system) influence evidence use in decision-making on innovation. At the professional level, preferences vary by professional group and health service setting. Commissioners favoured evidence derived from contact with colleagues or professional ‘networking’, combined with service user involvement and assessment of local needs rather than research evidence. Doctors in acute settings preferred research evidence, while those working in primary care emphasised clinical and experiential knowledge of patients’ needs. Preferences for non-research evidence were partly due to barriers to using some forms of research, e.g. cost analyses, or a perceived lack of formal evidence for making the decision at hand. Professional interests, and dynamics of power between professional groups, shaped the construction, interpretation and application of evidence during decision-making on innovation. Organisational roles included influencing the culture of evidence use (e.g. encouraging decisions to be data-driven), assessing non-clinical aspects of evidence (e.g. financial impact of innovation) and enabling stakeholder involvement. At the local system level, the embedding of pan-regional organisations shaped innovation decision-making at lower levels, while external pressures could encourage particular types of evidence (e.g. cost analyses) or inhibited its use. The politics of decision-making, e.g. linked to the financial context in which innovations were being considered, was an important influence on evidence use at all levels. An overview of the themes identified is provided in Table 1.

**Table 1:** Overview of the themes identified through the systematic scoping review

Themes		
Professional level	Organisational level	Local system level
Preferences for evidence	Organisational roles	External pressures
• Varies by professional group and across health care sectors.	• Limit innovations where evidence lacking, assess finance and budgetary issues, and enable stakeholder involvement.	• Influenced how evidence was used in decision-making.
Professional interests	Organisational facilitators	Pan-regional organisations
• Influence professional groups’ preferences for innovations and responses to evidence.	• Being ‘data-driven’, well informed to take risks, strong leadership and structures for stakeholder involvement.	• Downward influence on evidence use in local decision-making.
		• Upward relationship whereby pan-regional organisations legitimised innovations/encourage disinvestment at organisational level
Power dynamics	Organisational barriers	Widening stakeholder involvement:
• Choice of evidence, its interpretation and use in adoption decisions negotiated.	• Time, resources and pressures; authority to implement change; centralised approach to decision-making.	• External networks enable wider range of potential stakeholders to inform decision-making.
	Organisational politics	
	• Shapes selection and interpretation of evidence.	

### Multi-level interactions and their influence on evidence use

**Fig. 2:**
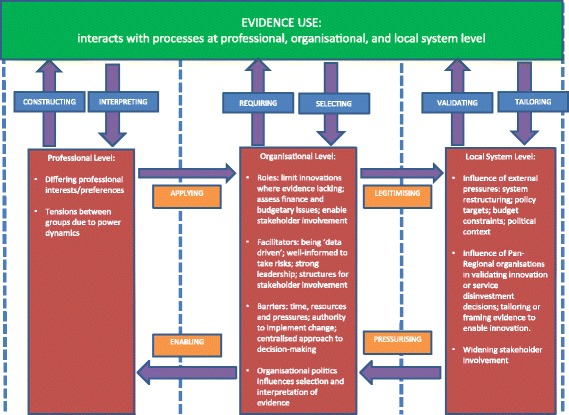
Interactions between evidence use and processes at different contextual levels (professional, organisational, local system). At the professional level, evidence is constructed and interpreted by members of professional groups. Professional groups can have differing preferences, self-interests and power relationships with other groups with regard to the use of evidence in decision-making. At the organisational level, organisations establish requirements for evidence to support decision-making and select evidence for informing decisions. Organisations have a number of roles in enabling evidence use; organisational barriers, facilitators and politics may shape the incorporation of evidence in decision-making. At the local system level, evidence is validated (e.g. endorsed by pan-regional bodies) and results are tailored to different local groups and organisations. Pan-regional groups can widen stakeholder involvement in decision-making. There are interactions between levels: professional groups *apply* evidence at the organisational level, while organisations *enable* professions to access and use evidence; organisations use local systems’ views on evidence to *legitimise* innovation or service disinvestment; and local system processes place *pressure* on the use of evidence for innovation (e.g. signalling the need for innovation or service disinvestment)

Much of the existing literature on evidence use in decision-making on innovation has focussed on processes at a particular level or not been explicit about the need to study processes at different levels (a notable exception is Prosser and Walley’s study [50] of the ways in which a primary care organisation attempted to influence the prescribing strategies of local GPs). Our synthesis of the current literature instead suggests the importance of the metaphor of a ‘system’ or ‘ecology’ to encompass the multi-level influences on evidence use in decisions about innovation. The importance of interactions between levels in influencing evidence use has emerged from our meta-case analysis of the synthesised literature. A map of processes at each level, and influence of the interactions between levels, is presented in Fig. 2. Adopting a multi-level perspective develops diffusion of innovations theory in two ways. Firstly, the decision-making agent is often more diffuse than the individual unit identified in current theory. Multiple stakeholders, including different professional groups, provider organisations and local system intermediaries, can inform adoption decisions collectively, particularly in relation to major system change in health care. Secondly, the analytical distinction found in diffusion of innovations theory between evidence, on the one hand, and decision-making agent on the other, should be reconsidered to account for the ways in which these phenomena are mutually defined (e.g. evidence informs decision-making when mobilised by health professionals, organisations or local system intermediaries, while such agents draw on different types of evidence to engage with and exert influence on decision-making).

### Implications for research

The review suggests implications for how evidence use in decisions about innovation is studied by researchers. Despite critiques of EBM emerging since the mid-2000s, rationalist conceptions of evidence based on this approach continue to inform many primary studies of evidence use in decision-making. This is apparent in discussions by researchers of ‘hierarchies’ of evidence, where research evidence is still privileged relative to other forms of information or ways of knowing. In such studies, endorsement of a hierarchy among different types of evidence may be implicit or explicit. For example, Evans et al. [17] were critical of the lack of use of ‘high-grade’ research evidence by local commissioners on Welsh Health Boards (often due to political and budgetary pressures), highlighting the potential effect on patient care, outcomes and resource use where research evidence was lacking and decision-makers relied on local evidence. This conclusion reflects scholarship advocating EBM whereby the quality of ‘scientific’ evidence (using recognised and reproducible methods) should be prioritised over local, ‘colloquial’ evidence [33]. Others question the need for research to demonstrate quality according to EBM standards [34], with pluralistic analyses highlighted as one potential cost [64].

Rather than evaluate the ‘quality’ of evidence through an EBM frame which tends to privilege a clinical perspective and formal evidence of effectiveness [27], we suggest that other forms of evidence and stakeholder perspectives are recognised as contributing to decision-making on innovation in their own right and on their own terms. As the focus groups highlighted, this inclusive approach would reflect the burgeoning forms of evidence now available to decision-makers (e.g. non-health care industry evidence, patient stories, feedback from user groups, reuse of existing data, case studies, infographics, lay summaries and evidence to support implementation). We suggest that such evidence diversity places a responsibility on decision-makers to be explicit about the types of evidence on which decisions are made, the stakeholder perspectives represented and any areas of uncertainty where evidence is lacking or inconclusive. Improvement work by researchers could focus on developing an explicit framework–which includes guidance on judging diverse evidence and stakeholder mapping–to support this activity. This would allow practitioners to consider whether sufficient stakeholder perspectives, and evidence reflecting those, are adequately represented in decision-making on innovations that often affect multiple groups, especially major system change [65].

While the review found that research evidence was the most cited form of information used in decision-making, three-quarters of the studies also referred to other forms of evidence, including local data and professional experience. Thus, studies at both local and policy level indicate the importance of ‘informal’ information [35]. Further qualitative research on practices of decision-making that highlights how and why different types of evidence come to count during decisions, and tracks the political aspects of decisions about innovation, would be fruitful (e.g. how the validity of evidence is constructed, why some forms of evidence might be prioritised and others marginalised and which professional, organisational and system level interests were influential). In existing research, the ‘decision-maker’ responsible for making decisions about innovation is often unclear. Future studies should be explicit about the approach to decision-making taken, how stakeholders were involved, e.g. distinguishing between decision-‘makers’ and decision ‘influencers’ [37], and how decision-making processes influenced adoption decisions.

### Strengths and limitations

In contrast to systematic reviews, some argue that the need to formally assess the methodological quality of studies in scoping reviews is relaxed [66]. However, we suggest this review was strengthened by the quality assessment of the included studies, as an objective was to provide recommendations for policy and practice that were based on robust studies. A further strength of this review was the inclusion of stakeholder feedback on the findings, meaning that we were able to test the practical relevance of the themes identified against ‘real-world’ accounts of decision-making on innovation. It is acknowledged that the focus groups were conducted at a time of significant concern about NHS funding. Nonetheless, the focus groups highlighted the importance of the financial aspects of innovations; information that showed innovations would reduce costs or be cost neutral was a priority when assessing new and existing innovations, confirming a concern with the financial impact of innovations in more recent literature published since the financial crises [7, 17, 59, 60]. The focus groups suggested that evidence use in decisions about service disinvestment should be disentangled from the broader concept of ‘innovation’ or ‘improvement’. In future research, the attributes and impact of innovations should be clearly defined to avoid forms of change due primarily to financial constraints being associated uncritically with the positive connotations of the term innovation.

The results of the database search indicated that some relevant papers were missing, based on the authors’ prior awareness of the field to develop the wider study protocol [40]. The manual search produced 8/31 included studies; a suggested reason for this relatively high number is that some terms used to describe innovation or improvement were not included in the database search, e.g. service development, planning, redesign and transformation. An additional manual search of selected management and health policy journals, books and grey literature was undertaken which included these terms; bibliographies of recent and highly relevant papers were also consulted.

## Conclusions

The synthesis of results from primary qualitative studies showed that evidence use in decision-making on innovation is influenced by processes at multiple levels. Moreover, our reading of the synthesised literature suggests that interactions (upwards and downwards) between conceptual levels shape evidence use in decision-making (e.g. professional groups can use local systems to legitimise innovations, while local systems can frame evidence in particular ways to influence activity at lower levels). We conclude with recommendations for policy and practice in terms of enhancing the use of evidence in decisions about innovation. First, while a range of evidence may inform decision-making, from research evidence through to local data and professional opinion, key decision-makers should reflect on the types of evidence that are routinely used in decision-making and how this influences the outcome (e.g. how might a preference for local data over research evidence contribute to the perceived risk of introducing innovations?). Second, the role of politics and power in decision-making needs to be acknowledged and skilfully managed. Evidence can potentially have an emancipatory role in lending authority to participants beyond other characteristics (e.g. personal credibility and positional power). To enable this role, organisations need to value challenging evidence and perspectives and build staff and organisational capacity in acquiring and applying evidence. Third, decision-makers need to consider the ways in which the environment in which decisions are made encourages diverse evidence and perspectives. For example, organisational leaders should consider how to mitigate professional interests and power when developing processes for enabling stakeholder involvement in decision-making.

## Additional files

Additional file 1: PRISMA checklist. (DOC 59 kb)

Additional file 2: Summary of search strategies. (DOCX 97 kb)

Additional file 3: Characteristics of primary studies included in full text review and quality assessment [6–12, 17, 47–63, 67–72]. (DOCX 29 kb)

Additional file 4: Charting of themes across primary studies included in full text review [6–12, 17, 47–63, 67–72]. (DOCX 40 kb)

Additional file 5: Focus group reporting. (DOCX 115 kb)

## Abbreviations

CASP: Critical Appraisal Skills Programme
CHF: Congestive heart failure
CLAHRC: Collaborations for Leadership in Applied Health Research and Care
COREQ: Consolidated criteria for reporting qualitative research
EBM: Evidence-based medicine
GP: General practitioner
HMIC: Health Management Information Consortium
MeSH: Medical Subject Headings
NHS: National Health Service
OECD: Organisation for Economic Co-operation and Development
PAG: Project advisory group
