# Supplementary material for: Evidence use in decision-making on introducing innovations: a systematic scoping review with stakeholder feedback
Source: Implement Sci. 2017 Dec 4;12:145. doi: 10.1186/s13012-017-0669-6 (PMC5715650; doi:10.1186/s13012-017-0669-6)
Supplement: Supplementary file 2 — Summary of search strategies. (DOCX 97 kb) [file 13012_2017_669_MOESM2_ESM.docx]

| **Summary Table**. Registry of the Bibliographic Searches***.*** | | | | | | |
| --- | --- | --- | --- | --- | --- | --- |
| **Databases** | **Platform/ Access** | **Search date** | | **References retrieved** | |  |
| Medline | Ovid Licensed Resource | 26/05/2016 | | 649 | |  |
| EMBASE | Ovid Licensed Resource | 24/05/2016 | | 753 | |  |
| PsycINFO | Ovid Licensed Resource | 24/05/2016 | | 128 | |  |
| Scopus | Elsevier Licensed Resource | 24/05/2016 | | 319 | |  |
| HMIC Health Management Information Consortium | Ovid Licensed Resource | 24/05/2016 | | 185 | |  |
| EBSCO Business Source Complete | EBSCO Licensed Resource | 24/05/2016 | | 86 | |  |
| **TOTALREFERENCES** |  |  | **2120** | |  |  |
| **REFERENCES (Duplicates removed)** | |  | **1816** | |  |  |
|  | |  |  | |  |  |

**Search strategies**

|  |  | **Medline (WoS)** Date: 26 May 2016 |  |
| --- | --- | --- | --- |
| **Blocks** |  | **Search terms** |  |
|  | #15 | Remove duplicates from #14 |  |
|  | #14 | #4 and #6 and #13 |  |
|  | #13 | Limit #12 to (english language and yr="2000 -Current") |  |
|  | #2 | exp Organizational innovation/ or exp diffusion of innovation/ or exp inventions/ or exp health care reform/ or exp quality improvement/ or exp translational medical research/ or (innovation or improvement).ti,ab. |  |
|  | #11 | remove duplicates from #10 |  |
|  | #10 | #4 and #6 and #9 |  |
|  | #9 | #2 or #8 |  |
|  | #8 | limit #7 to (english language and yr="2000 -Current") |  |
|  | #7 | (innovation or improvement).ti,ab. |  |
|  | #6 | limit #5 to (english language and yr="2000 -Current") |  |
|  | #5 | exp Decision making/ or exp decision making, organizational/ or exp clinical decision-making/ or exp decision theory/ |  |
|  | #4 | limit #3 to (english language and yr="2000 -Current") |  |
|  | #3 | exp Evidence-based practice/ or exp evidence-based medicine/ or exp evidence-based nursing/ or exp management audit/ or exp knowledge management/ or exp knowledge bases/ or exp knowledge/ or exp public opinion/ |  |
|  | #2 | limit #1 to (english language and yr="2000 -Current") |  |
|  | #1 | exp Organizational innovation/ or exp diffusion of innovation/ or exp inventions/ or exp health care reform/ or exp quality improvement/ or exp translational medical research/ |  |

|  |  | **EBSCO Business Source Complete** Date: 24 May 2016 |
| --- | --- | --- |
| **Blocks** |  | **Search terms** |
|  | #6 | #1 AND #2 AND #3 AND #4. **Limiters** - Published Date: 20000101-20160531; Document Type: Article; Language: English |
|  | #5 | #1 AND #2 AND #3 AND #4 |
|  | #4 | TI ( health care or healthcare ) OR AB ( health care or healthcare ) OR SU ( health care or healthcare ) |
|  | #3 | TI ( decision* or decision making ) OR AB ( decision* or decision making ) OR SU ( decision* or decision making ) |
|  | #2 | TI evidence OR SU evidence OR AB evidence |
|  | #1 | TI ( innovation or improvement ) OR AB ( innovation or improvement ) OR SU ( innovation or improvement ) |

|  |  | **PsycINFO** Date: 24 May 2016 |  |
| --- | --- | --- | --- |
| **Blocks** |  | **Search terms** |  |
|  | #9 | #2 and #4 and #6 and #8 |  |
|  | #8 | limit #7 to (english language and yr="2000 -Current") |  |
|  | #7 | (health care or healthcare or health services).ti,ab. |  |
|  | #6 | Limit #5 to (english language and yr="2000 -Current") |  |
|  | #5 | decision*.mp. or decision-making.ti,ab. [mp=title, abstract, heading word, table of contents, key concepts, original title, tests & measures] |  |
|  | #4 | limit #3 to (english language and yr="2000 -Current") |  |
|  | #3 | evidence.ti,ab. |  |
|  | #2 | limit #1 to (english language and yr="2000 -Current") |  |
|  | #1 | (innovation or improvement).ti,ab. |  |

|  |  | **HMIC** Date: 24 May 2016 |  |
| --- | --- | --- | --- |
| **Blocks** |  | **Search terms** |  |
|  | #7 | #2 and #4 and #6 |  |
|  | #6 | limit #5 to yr="2000 -Current" |  |
|  | #5 | decision*.mp. or decision-making.ti,ab. [mp=title, other title, abstract, heading words] |  |
|  | #4 | limit #3 to yr="2000 -Current" |  |
|  | #3 | evidence.ti,ab. |  |
|  | #2 | limit #1 to yr="2000 -Current" |  |
|  | #1 | (innovation or improvement).ti,ab. |  |

|  |  | **EMBASE** Date: 24 May 2016 |  |
| --- | --- | --- | --- |
| **Blocks** |  | **Search terms** |  |
|  | #9 | #2 and #4 and #6 and #8 |  |
|  | #8 | limit #7 to (english language and yr="2000 -Current") |  |
|  | #7 | (healthcare or health care or health services).ti,ab. |  |
|  | #6 | limit #5 to (english language and yr="2000 -Current") |  |
|  | #5 | (decision* or decision-making).ti,ab. |  |
|  | #4 | limit #3 to yr="2000 -Current" |  |
|  | #3 | evidence.ti,ab. |  |
|  | #2 | limit #1 to (english language and yr="2000 -Current") |  |
|  | #1 | (innovation or improvement).ti,ab. |  |

|  |  | **SCOPUS** Date: 24 May 2016 |  |
| --- | --- | --- | --- |
| **Blocks** |  | **Search terms** |  |
|  | #2 | #1 LIMIT-TO ( LANGUAGE , "English" ) ) |  |
|  | #1 | TITLE-ABS ( evidence AND decision* AND innovat* AND health* ) |  |
