# Supplementary material for: Evidence use in decision-making on introducing innovations: a systematic scoping review with stakeholder feedback
Source: Implement Sci. 2017 Dec 4;12:145. doi: 10.1186/s13012-017-0669-6 (PMC5715650; doi:10.1186/s13012-017-0669-6)
Supplement: Supplementary file 3 — Characteristics of primary studies included in full text review and quality assessment [6–12, 17, 47–63, 67–72]. (DOCX 29 kb) [file 13012_2017_669_MOESM3_ESM.docx]

**Characteristics of primary studies included in full text review and quality assessment. Greyed out references were not included in the thematic analysis [6–12, 17, 47–63, 67–72]**

| **Reference** | **Setting (country; acute or primary care provision, commissioning)** | **Type of Innovation/ Service Improvement** | **Type(s) of Evidence** | **Quality Assessment Score out of 9** |
| --- | --- | --- | --- | --- |
| Ahmad et al. (2012) [10] | UK; Acute and Primary Care | Infection prevention and control technologies (covering environmental hygiene; catheter care; medical devices hygiene; diagnostics; hand hygiene; information management  and communication; patient hygiene; training). | - Staff involvement in implementation, with some organisations prioritising ‘specialist’ knowledge of IPC team. - Staff understanding of ‘patient perceptions’ (e.g. number of complaints as ‘soft data’) | 6 |
| Armstrong et al. (2013) [12] | UK; Acute and Primary Care | Three case study quality improvement projects | - Role of ‘patient and public involvement’ in quality improvement; whether and how patients can be involved in distinctive and meaningful ways relative to professional roles and knowledges. | 8 |
| Bouwman (2008) [47] | Denmark, Ireland, The Netherlands, Slovenia,  Spain, Sweden, Switzerland and Australia; Primary care | Gene-based personalised nutrition advice | - Nutritional studies | 5 |
| Bowen et al (2009) [54] | Canada; Commissioning | Regional health authority planning | - Little consensus on definition of evidence; often assumed to be limited to ‘research’ or ‘quantitative data’. | 6 |
| Carstens (2009) [58] | United States; cross-cutting systems of care covering mental health, social welfare, justice, education, and other  child-serving agencies. | Multi-Systemic Therapy | - Evidence that multi-systemic therapy improves clinical outcomes for both children and families and also enhances system performance. | 9 |
| Challans (2006) [67] | UK; Commissioning | Service improvement | - Patient involvement as form of knowledge | 0 |
| Checkland (2007) [55] | UK; Primary Care | Models of clinical management and service delivery for a variety of chronic diseases | - National Service Frameworks (policies) | 8 |
| Danjoux et al (2007) [52] | Canada; Acute Care | New technology for repair of abdominal aortic  aneurysms (endovascular aneurysm repair [EVAR]) | - Adoption based on surgeons’ perceptions of improved patient outcomes and safety (however, only preliminary data on safety and effectiveness existed). - Decision to stop funding based on cost, lack of evidence of safety and effectiveness and the fact that vascular surgery was not a local priority at the time. | 7 |
| Evans et al. (2013) [17] | UK; Commissioning | National chronic conditions management policy | - Research evidence one among many influences: variety of information sources informed decision-making; ‘high-grade’ research evidence (e.g. systematic reviews) lacking in decision making. Evidence of local need important. - Rather than being seen by participants as research evidence, evidence defined as information derived from contact with colleagues or professional ‘networking’, involving service users, and assessing local needs (idea of ‘responsive practice’). | 8 |
| Gallego (2008) [7] | Australia; Commissioning | New health technologies at the regional and institutional level | - Economic evaluation | 7 |
| Harden and Fulop (2015) [61] | Australia; Acute Care | Multi-disciplinary cancer care | - Knowledge as ‘fact’, ‘expert opinion’, and ‘narrative’ used in decision-making conversations. - Lack of ‘narrative’ evidence used by the networks, as factual and expert talk often dominated. | 7 |
| Hendy and Barlow (2013) [53] | UK; Health and social care organisations | Remote care (telecare) services | - Experiential knowledge, e.g. stories (actual practice) - RCT / clinical trial / cost-effectiveness (aspiration) - Internal, e.g. questionnaires completed by front-line staff | 7 |
| Hutchinson and Johnston (2008) [68] | Australia; Acute Care | Clinical management tools | - Involved use of practical knowledge (e.g. clinical scenarios with patients), professional opinion (e.g. ‘what worked best’), contextual knowledge, and research evidence (latter was limited). | 5 |
| Kyratsis (2012) [49] | UK; Acute and Primary Care | Infection Prevention and Control technologies | - Different types of innovation knowledge: Awareness knowledge (information that an innovation exists); ‘how-to’ knowledge (information required to use an innovation properly at individual and organisational levels); ‘principles’ knowledge (information about an innovation’s functioning principles). | 8 |
| Kyratsis et al (2014) [6] | UK; Acute Care | Specific technology examples | - Evidence types included research-generated information on innovation decisions from national bodies and agencies, local trial data, peer exchange or, less often, input from external agents such as management consultants. - ‘Evidence templates’ shape evidence use, including: ‘biomedical-scientific’ (thorough testing); ‘practice-based’ (learning from others); and ‘rational-policy’ (policy requirements). | 7 |
| Lettieri (2009) [69] | Italy; Acute Care | Technology assessment at the hospital level | - Evaluations used to make adoption decisions. Assessment of uncertainty partial and variable relative to assessment suggested by literature. Approaches include using scenario analysis, sensitivity analysis, and expected variability of clinical practice. | 5 |
| Lopes et al (2015) [62] | Australia; Commissioning | New health technologies | - Stakeholders conceptualised and valued evidence differently, from clinical outcomes and patient preferences to patients' experiences in living with illness. | 8 |
| Mele et al. (2013) [56] | Italy; Acute Care | Technological innovation (Da Vinci surgical robot) | - Prevailing types of evidence vary by region, ranging from scientific evidence to experiential knowledge. | 6 |
| Nedlund and Garpenby (2014) [51] | Sweden: Acute and Primary Care | New health technology at the regional level | - Scientific studies (awareness that technologies could not always be evaluated using criteria used in randomised controlled trials). | 7 |
| Nembhard (2015) [11] | United States; Acute Care | Quality improvement in the care of patients experiencing heart attack | - Staff voice in decision-making | 7 |
| Noël et al. (2014) [70] | United States; Primary care | Chronic Care Model | - Practice facilitation, including evidence-based ‘toolkit’. | 4 |
| Panzano and Roth (2006) [71] | Canada; Mental health Coordinating Centres of Excellence | Four innovative mental health practices (cluster based planning/multi-systemic therapy/the Ohio medication algorithms related to schizophrenia and depression/integrated dual disorder treatment) | - Scientific evidence and experiential evidence | 4 |
| Prosser and Walley (2007) [50] | UK; Primary care | Primary care group/trust prescribing strategies | - Development of formularies, educational outreach, prescribing feedback, dissemination of drug information, educational meetings, and peer group review. | 7 |
| Richer et al. (2013) [72] | Canada; Acute Care | Major organisational transition | - Participants identified range of evidence: historical and local data, best practices, benchmarking with other organizations, and literature review with quantitative and qualitative studies. | 5 |
| Robert et al. (2011) [63] | UK; Acute Care | National quality improvement programme: The  Productive Ward in England. | - Programme led by a national body, the National Health Service Institute for Innovation and Improvement (NHSI) in England. | 7 |
| Rycroft-Malone et al. (2013) [57] | UK; Acute Care | Evidence based guideline recommendations for reducing peri-operative fasting times | - Research evidence in the guideline (‘strong’, underpinned by RCTs); patient guideline; facilitation (championing, awareness raising, role modelling); local evidence and practitioner experience. | 8 |
| Spyridonidis et al. (2011) [60] | UK; Acute and Primary Care and Commissioning | In response to guidelines for obesity and CHF, introduction of changes to prevent obesity and a community CHF service respectively. | - National guidelines (NICE). - Local guidelines and dissemination plans (face-to-face meetings, educational and teaching workshops). | 8 |
| Teng et al. (2007) [9] | Canada; Commissioning | Priority setting by a provincial health authority | - Evidence use in priority setting perceived to need improvement (currently ad hoc and more based on ‘whoever yells the loudest’). - Suggested improvement strategies include: decision-making criteria (inconsistent); ‘best practice information’; creation of ‘Strategic Plan’; stakeholder opinion (including public opinion). | 7 |
| Wade et al (2016) [59] | Australia; Primary care | Telehealth in the home | - Published evidence of benefits from trial evaluation. | 9 |
| Williams and Bryan 2007 [8] | UK; Acute and Primary care | Medical technologies | - Cost-effectiveness analysis information | 8 |
| Wye et al (2015) [48] | UK; Commissioning | Commissioning | - Wide range of sources. Local data often preferred to national or research-based information. Barriers to use of academic research, which had a lesser role in decision making. | 9 |
