# Supplementary material for: Evidence use in decision-making on introducing innovations: a systematic scoping review with stakeholder feedback
Source: Implement Sci. 2017 Dec 4;12:145. doi: 10.1186/s13012-017-0669-6 (PMC5715650; doi:10.1186/s13012-017-0669-6)
Supplement: Supplementary file 4 — Charting of themes across primary studies included in full text review [6–12, 17, 47–63, 67–72]. (DOCX 40 kb) [file 13012_2017_669_MOESM4_ESM.docx]

**Charting of themes across primary studies included in full text review. Greyed out references were not included in the thematic analysis [6–12, 17, 47–63, 67–72]**

| **Reference** | **Study type and methods** | **Aims and objectives** | **Professional processes** | **Organisational processes** | **Local system processes** |
| --- | --- | --- | --- | --- | --- |
| Ahmad et al. (2012) [10] | Multiple case study design; thirty-eight technology adoption decisions and implementation processes across 12 NHS organisations  (121 interviews). | To investigate innovation adoption decisions and implementation processes from an organisational perspective, particularly stakeholder involvement. | - Credibility of senior clinicians (e.g. medical director) used to legitimise decision making on innovation. | - Early engagement of frontline clinical staff and technology  users in decision-making aids implementation (however, some organisations took ‘exclusive’ approach limed to the central infection prevention and control (IPC) team as perceived to hold necessary ‘specialist’ knowledge). | - |
| Armstrong et al. (2013) [12] | Ethnography; case studies of three quality improvement projects within the ‘Closing the Gap through  Clinical Communities’ programme (‘Lung Cancer’, ‘Aneurysm’, and ‘Kidney’ projects). Data collected using non-participant observations, 126 interviews, documentary analysis. | To characterise patient involvement in three improvement projects and identify strengths and weaknesses of contrasting approaches. | Patient representatives rely on  broader knowledge, skills and experiences to contribute and have potential roles as ‘persuader’ and ‘knowledge broker’.  However, when  discussion focused on technical issues (e.g.  specifications for equipment standards), it was  less clear how patients could contribute. Clinicians  doubted patients’ ability to  contribute to technical and safety issues, questioning their knowledge/experience. | Meaningful involvement is supported by early involvement in the project, effective communication channels, creation of a non-hierarchical structure, and clearly defined patient roles. | - |
| Bouwman (2008) [47] | Qualitative; 15 GPs were interviewed to collect their perceived barriers and opportunities  towards involvement in gene-based nutrition advice. | To explore the issues that facilitate  or hinder the involvement of GPs in an early stage of  the development process of innovative, personalized  nutrition advice. | Most GPs contested the results of nutritional studies. GPs’ arguments against gene-based nutrition advice related to (1) little knowledge of field (i.e. General Practice), (2) relevance and quality of evidence, (3) perceived needs of patients. | - | Findings suggest need for early involvement of GPs in development process (e.g. in order to provide expertise about patients). |
| Bowen et al (2009) [54] | Qualitative study involving planners and decision-makers in 11 health authority regions. 17 focus groups and 53 interviews with managers (205 participants in total). | Explore views on the nature and use of evidence, and barri­ers to making decisions based on evidence. | - Research capacity and data availability less important relative to political and organizational factors; suggestion that evidence could be ‘gamed’ | - Lack of time and resources (more resources could be allocated if considered organisational priority).  - Centralised decision-making and lack of communication inhibited evidence use.  - Crisis management ‘culture’, excessive workload, fracturing of attention | Majority of barriers seen as ‘external’. Politics often more important than evidence, e.g. reactions to public perceptions |
| Carstens (2009) [58] | Mixed methods study of 13 systems of care; qualitative key informant interviews (n=39) and follow up quantitative surveys. | To analyse decision making about the adoption  and implementation of evidence-based practices within local systems of collaboration. | Entrepreneurial leaders of adopter sites suggested that they made more evidence led decisions (e.g. well informed on research and data) than those in non-adopter sites. | EBP seen by leaders as way of increasing agencies’ competitive advantage. | Adopting EBP understood as way of enhancing local system legitimacy, e.g. to attract public funding. |
| Challans (2006) [67] | Case study of clinical audit patient panel. Information provided by Sheffield South West Primary Care Trust, England. | To identify the ways in which patients  can be involved in service improvement and the ways  in which they are able to contribute to improvement agenda. | Staff initially apprehensive about involving patient panel members in the project team. Patient involvement requires change in culture for some health care professionals. | - | - |
| Checkland (2007) [55] | Case studies of four general medical practices based on interviews (n=36), non-participant observation and documentary analysis. | To investigate how general medical practices in the NHS react to a mandated external initiative,  National Service Frameworks (NSFs), and to explore the value of using ‘barriers to change’ for understanding this. | Failure to implement NSF were linked by participants to concerns about NSFs as a form of evidence (e.g. document length, complexity, local applicability), but the authors suggest these were constructions that were used by GPs because the NSFs did not fit in with their ‘identity work’. | Non-implementation  of policy was related to underlying organisational issues. | - |
| Danjoux et al (2007) [52] | Qualitative case study and evaluation; interviews (n=5) with those leading a surgical innovation and documentary analysis. | To describe and evaluate the  adoption of a new health technology used by surgeons for  the treatment of aortic aneurysms (endovascular  aneurysm repair). | Surgeons’ desire to introduce  new techniques and innovative approaches that ‘make sense’ for the patient (the “medical-individualistic”  perspective). | Innovation encouraged as an academic health science centre.  Cost and lack of evidence of safety and effectiveness informed decision to cut funding. | Ontario's Ministry of Health and  Long-Term Care recommended against adoption (citing need for long-term follow-up data from clinical trials), influencing hospital’s decision to cut funding. |
| Evans et al. (2013) [17] | Mixed methods; national email survey of health service commissioners in Local Health Boards (n=22) and semi-structured interviews (n=5). | To understand the role of research evidence at the local level in relation to the  implementation of a national chronic conditions management policy. | According to one senior manager, preference for approach to care ‘informed by professional contacts’ over other forms of evidence. | Tension between resources required to appraise research to inform commissioning  decisions and that required to implement changes to services. | Government policy, initiatives, and targets influenced commissioning context and drove decision making.  Evidence-based  policy valued but its use in practice constrained by budgetary pressures. |
| Gallego (2008) [7] | Qualitative study of decision-makers’ perceptions; interviews (n=12) with senior managers, clinical service (medical), middle managers, medical  clinicians, and nurse managers. | To aid the design of a new process of technology assessment and decision making. | Some decision-makers lack knowledge and understanding of economic evaluation; its credibility and accuracy, especially at a local system level, questioned. |  | Decision-makers’ narrow view of economic evidence - based on costs and budgetary constraints – influenced potentially by need for rationing in health care system (budgetary impact and costs main deciding factor). |
| Harden and Fulop (2015) [61] | Qualitative study of seven Cancer networks, responsible for enhancing multi-disciplinary cancer care. Data collected through video-recordings of fifty-three network sub-committee meetings. | To explore how decision-making can be improved in healthcare contexts, such as cancer care networks, by adopting ‘relational’ leadership  practices. | Committee chairs’ moderate use of different types of evidence (leadership) (what the authors term the difference between ‘single ontology’ and ‘multi-ontology’ sense making). | Institutional or management issues in the organisation of care steered conversations toward scientific and technical themes at the expense of narrative perspectives, representing ‘single ontology’ sense-making. | - |
| Hendy and Barlow (2013) [53] | Ethnography; five comparative, longitudinal case studies of remote care (telecare) services using formal interviews (115 hours), informal discussions and meetings (41 hours), observations (70 hours), and documentary analysis. | To explore how managers' use evidence to inform decisions about innovation adoption. | - Evidence adapted to benefit managers and local staff based on managers’ agendas (e.g. alignment with existing practices and needs, moderating innovation). | - Innovation spread mediated by its alignment with recipient organisation (non-alignment of organisation's values and expectations and managers' agendas in 2/5 cases). | - |
| Hutchinson and Johnston (2008) [68] | Qualitative, non-participant, observational design. Two multidisciplinary teams’ meetings observed (n=7) and interviews with participants (n=10). | To investigate the process of evidence use by  health professionals during development of evidence-based clinical management  tools. | Multidisciplinary meetings were dominated by doctors’ professional opinions, while those of allied health professionals and nurses were less able to influence tool development. | - | - |
| Kyratsis (2012) [49] | Qualitative, multisite, comparative case study design,  individual and group interviews (n=121) and observations (20 hours). | To understand organisational technology adoption (initiation, adoption decision,  implementation) by looking at the different types of  innovation knowledge used during this process. | - Professional networks important source of three knowledges.  - Preferences varied by professional group. Nurses used both ‘principles’ (scientific) and ‘how-to’ knowledge’; medical professionals prioritised ‘principles’ knowledge. | Research active organisations sought and prioritised ‘principles’ knowledge.  - Need for clinical and financial justification for innovations. | - |
| Kyratsis et al (2014) [6] | Comparative mixed methods case studies of 27 technology product journeys within nine acute NHS Trusts. Data collected using surveys, in-depth interviews (n=191) and documentary analysis. | To investigate the use of research-based knowledge in health care management decisions about innovation. | Managers with different professional backgrounds sought and used different forms of evidence in decision-making, based partly on ‘plausibility to self’.  Doctor managers and non-clinical managers were concerned with evidence that helped their own decision-making, whereas nurses were  also concerned with providing evidence to aid others’ decision-making. | Access and use of evidence in decision-making aided by organisational processes, e.g. infrastructure redevelopment projects and emphasis on patient safety, collaboration or  teamwork (i.e. through organisational culture). | External pressures and critical events, e.g. national performance targets and financial pressures, influenced decision-making. Encouraged more emphasis on ‘what works’ than rigorous evidence. |
| Lettieri (2009) [69] | Multiple case study on current practice of technology assessment in 5 hospitals using interviews (n=15) and documentary analysis. | To assess the extent to which and how uncertainty is taken into account for budgeting technology adoption at a hospital level. | Sponsors of new technologies were often doctors who understood the clinical case for particular innovations, but were less confident with organisational and financial issues, suggesting a need for other stakeholders to use evidence to assess these issues. | Suggests organisational ways of managing uncertainty related to technology adoption, including building evidence based practice for technology selection and a reporting system regarding technology performance to inform future decision making. | - |
| Lopes et al (2015) [62] | Qualitative interview study (n=13) | To explore the views of patient organisation representatives and members of Advisory Committees providing advice to the Australian Department  of Health (DoH) on decisions related to public funding for  new health technologies. | Mismatch between conceptions of useful evidence used by advisory committee (disease) and patient organisations (lived experience of illness), hindering involvement. | Involvement processes for including patient organisations  in health care funding decisions inadequate. Patient organisations partnered with other stakeholders to e.g. increase influence on policy making. | Suggest need for ‘deliberative’ involvement process with multiple stakeholders to make decision making more inclusive and transparent, while recognizing power dynamics. |
| Mele et al. (2013) [56] | Qualitative, multiple case study design. Data collected via interviews (n=148) and documentary analysis. | To explore the role of evidence in governing the adoption of technological innovation (Da Vinci surgical robot) in health care. | Managers use evidence to decline unreasonable requests from clinicians. | - | Four archetypes of regional decision-making based on policymakers’ preferences found: ‘competency network’ (research evidence); ‘authorization’ (secondary data, e.g. health technology assessment); ‘incentive’ (technical knowledge and monitoring), ‘central planning’ (experiential judgements and monitoring). |
| Nedlund and Garpenby (2014) [51] | Qualitative case study of Health Technology Advisory Committee (HTAC) based on interviews (n=19). | To shed light on how problem frame differences on evidence based policy (EBP) in a regional healthcare context, shape the puzzling over how to handle the influx of new technologies. | Evidence given different meanings and problem frames by different individuals (e.g. “some of the actors would suggest that ‘‘a lack of evidence’’ was the problem, while other actors related to a situation where the available information was not underpinned by ‘‘good’’ evidence or that the introduction of new technology had been founded on limited evidence”). |  | Unit managers suggested HTAC was not embedded in the ordinary decision-making structures. Unit managers often preferred other solutions e.g. using professional reference groups and other professional and scientific networking groups. |
| Nembhard (2015) [11] | Qualitative study of staff perceptions in 12 hospitals using individual and group interviews (n=99). | To examine the drivers of voice for health professionals in hospitals. Specifically, to investigate the factors  that influence their voice, why these factors are influential, and the purposes for which staff use their voice. | Staff willingness to voice influenced by individual’s personality and perceived expertise (e.g. tenure), and availability of data to provide authority or legitimacy (e.g. performance data, benchmarking data, or national guidelines). | Leader supportiveness, organizational culture,  and structures supporting voice. | External validation of opinion voiced, e.g. participating in a national improvement campaign. |
| Noël et al. (2014) [70] | Mixed methods: cluster randomized controlled trial and ethnographic field notes recorded by the facilitators during ‘monthly facilitation meetings’ at practices over a 12-month period. | To examine the specific activities and Chronic Care model (CCM) components that primary care practices implemented and sustained in response to a 12-month Practice Facilitation (PF) intervention. | Practice staff were more likely to implement aspects of the model that were compatible with their own values, i.e. taking from the evidence the need to change patients’ behaviour, rather than their own. | The most popular interventions were simpler to implement and were proposed for a trial period reducing commitment among staff to implementing them beyond the short term. | Suggests more complex re-design require performance management, feedback, incentives. |
| Panzano and Roth (2006) [71] | Study focused on seventy-eight projects involving organisational decisions to adopt one of four innovative  mental health practices. Key informants provided information on the adoption decision via interviews and a survey (participant numbers not reported). | To examine the extent to which a risk-based decision-making framework is useful for understanding the decision to adopt research-guided, innovative mental health practices. | - | Organisations that are well informed about innovations, which extends to gathering information from peers, are more likely to adopt innovations due to a greater reported capacity to manage associated risks. | - |
| Prosser and Walley (2007) [50] | Qualitative study of stakeholders’ perspectives; data collected using focus groups (n=4) and  interviews (n=24) with GPs and others in primary care. | To examine key stakeholders’ perspectives on primary care prescribing strategies in context of managerial and  organisational changes in primary care at the time. | Managers privileged scientific,  evidence-based medicine, while marginalizing GPs’ clinical and experiential knowledge. GPs  sceptical of managers as objective decision makers and information providers (for GPs, clinical knowledge encompasses knowing the patient, too). |  | - in response to opposition from GPs, local primary care organisations (PCO) emphasised quality in prescribing targets rather than more controversial issue of cost containment.  - peer performance is an important influence on behaviour (e.g. data showing that practice an outlier). |
| Richer et al. (2013) [72] | Qualitative, single case study based on interviews (n=11) with key decision-makers across hospital. | To examine the  body of literature around notions of ‘evidence’  in the decision-making process. | Leaders relied on courage, an ability to ‘rally’ others around goals, and ‘displaying coherence’ between evidence and changes made. | Clinical basis for change considered first; however, political value/cost powerful influence on how evidence used in transformational change.  ‘Push’ (clear vision, guidelines, support) and ‘pull’ (incentives) factors needed to implement change. | The organizational,  social, and political context in which the organisation was at this time. |
| Robert et al. (2011) [63] | Mixed methods, including qualitative approach. Five organisational case studies of NHS acute hospitals, including 58 interviews. | To explore why innovations in service and delivery are adopted and how they are then successfully  implemented and eventually assimilated into routine nursing practice. | ‘Champion’ for programme and staff having practical need for change. | Enablers of adoption include solid financial footing; leadership and support from senior staff; local ownership and empowerment of staff; and resources to support innovation. | Source of evidence (NHSI) - had national organisational profile and established links with providers – which aided adoption. |
| Rycroft-Malone et al. (2013) [57] | Randomized controlled trial with embedded process evaluation based on interviews (n=139) and focus groups (n=5). | To provide an explanation of  implementation processes from one of the first national implementation research randomized controlled trials with embedded process evaluation conducted within acute care, and a proposed extension to the Promoting Action on Research Implementation in Health Services (PARIHS) framework. | Evidence base believed to be robust and was relatively uncontested. However, research base mediated by practitioner and patient judgements about the need for caution, and perceived attitudes to risk taking (difference between agreeing with evidence, and using it to make decisions and/or change  services). | Aligning implementation  with existing relevant activities enhanced the chances of  more successful implementation. | Study conducted at time of major NHS changes; staff reported feeling overwhelmed by competing priorities and managerial support variable. Success vested in individual’s enthusiasm and commitment. |
| Spyridonidis et al. (2011) [60] | Comparative, longitudinal  case-study design (74 interviews). | To inform ‘evidence-based’ implementation by using an innovative methodology to provide further understanding of the implementation process in the English NHS using two NICE clinical guidelines as exemplars. | Implementation influenced by doctors’ and managers’ receptivity; may engage or disengage with organisational initiatives for implementing new services in response to guidelines. | Implementation not ‘single decision’ but ‘numerous decision events’  The variations in the  implementation process could be best accounted for in terms of differences in the structure  and nature of the local organisational context. This points to the importance of  managers as well as clinicians in decision-making about implementation. | Financial incentives enhanced adherence to guidelines. |
| Teng et al. (2007) [9] | Qualitative study; 25 interviews with decision-makers in a provincial health services authority. | To assess how evidence is used in setting priorities by a provincial health authority, including organizational barriers and facilitators. | Stronger physician role needed, e.g. to provide and interpret clinical evidence (‘conflict of interest’ a barrier, as have fee-for-service model). | Organisational context influences decision-making processes; lack of authority to change process.  Strong leadership and commitment to priority setting needed.  Culture of ‘openness’, ‘learning’ and being ‘data-driven’ needed. | Politics influences decisions. |
| Wade et al (2016) [59] | Action research; data collected using semi-structured interviews (n=19) and ‘deliberative forum’ on preferred implementation models. | To produce a preferred implementation  approach for sustainable and large-scale operations, and a process model that offers practical advice for achieving  this goal. | Clinician acceptance, quoting interviewee: “one of the issues is having sufficient data to say this is a change that should be service wide” | Move from trials to large-scale services of home telehealth services still at early stage, requires leadership support to overcome variety of implementation barriers.  Leadership is enabled by 1) showing solutions  to the problems of service demand and budgetary pressure,  2) demonstrating how home telehealth aligns with health service policies, and 3) achieving clinician acceptance  through providing evidence of benefit and developing new models of clinical care. Change enabled by marketing telehealth to patients, clinicians and policy-makers, and building a community of practice. | Budgetary constraint at state and federal level meant that services demonstrating savings or efficiencies more likely to be funded. |
| Williams and Bryan 2007 [8] | Mixed-methods study. Qualitative  case studies of four decision-making committees including documentary analysis, observation of committee meetings (n=11), and interviews with committee members (n=31). Survey in primary and secondary care to collate information  proformas used by decision-making committees  when considering proposed new technologies. | To explore how  local committees operate when making technology coverage decisions, the information they use  and the extent to which economic evaluation  featured in this. | Other factors influenced the committees, such as the  perspectives of committee members, especially  clinicians. Some  respondents felt that the committees were susceptible to powerful personalities on, or attending, the committee. | In order to be useful, cost effectiveness analysis  needs to better reflect the constraints of the local decision-making environment.  Decision-making environment appeared to militate against emphasis on cost-effectiveness  analysis, including unclear relationships with resource allocators, an explicitly political decision-making  process, and poorly specified decision-making criteria. | - |
| Wye et al (2015) [48] | Qualitative study of four commissioning organisations in England, using interviews (n=52), meeting observation (n=14), and documentary analysis. | To identify the reasons that prompted commissioners to seek information, to clarify which sources and types of knowledge commissioners commonly consulted, and to describe the use of research  evidence in decision-making. | Commissioners acquired information through conversations, stories (clinical and patient) and documentation (especially bulleted summaries to capture attention) | -Competing proposals for funding (persuasion needed).  -Organisational processes change the original information. | Decisions need to stand up to external scrutiny (locally and nationally) |
