# Supplementary material for: Evidence use in decision-making on introducing innovations: a systematic scoping review with stakeholder feedback
Source: Implement Sci. 2017 Dec 4;12:145. doi: 10.1186/s13012-017-0669-6 (PMC5715650; doi:10.1186/s13012-017-0669-6)
Supplement: Supplementary file 5 — Focus group reporting. (DOCX 115 kb) [file 13012_2017_669_MOESM5_ESM.docx]

**Focus group reporting**

Reporting of the focus groups was informed by Consolidated criteria for reporting qualitative research (COREQ) checklist [45]. Focus group participants were identified using different channels. This included web-searches for examples of innovation in relation to the English NHS and inviting participants associated with these activities. The study’s project advisory group was utilised to identify potential participants. The focus groups, lasting two hours each, were structured using discussion topics derived from preliminary analysis of the literature review’s results (see below). The discussion topics were piloted with the study’s project advisory group and NIHR Collaboration for Leadership in Applied Health Research and Care (CLAHRC) North Thames’ research advisory panel. Participants read an information sheet and provided informed consent. Prior to the day, the participants were emailed a one-page document which gave an overview of the discussion topics and working definitions of key terms (e.g. organisational processes).

The participants were asked to come prepared to discuss a recent example of innovation that they had been involved in. The themes were discussed in relation to those innovations to examine their ‘real-world’ relevance and to identify any gaps in the literature review. The focus groups were facilitated by two researchers (ST and DD), with one researcher leading each discussion topic and the other asking follow-up questions or prompting as necessary. Participants were encouraged to have a group conversation and not just respond to the researchers. The discussions were audio-recorded and professionally transcribed. Thematic analysis was applied to the focus group transcripts mirroring the literature review’s themes; the resulting discussions were used to confirm or further develop the preliminary findings.

**Discussion guide for focus groups**

1. **Introductions and sharing examples of innovation [20 mins]**
   - Name
   - Your role and sector you work in (acute or primary care, commissioner or service provider, describe yourself as a clinician or manager, a patient representative, or another type of stakeholder)
   - Please describe a recent example of innovation or improvement you have been involved in. *For the purposes of this exercise, innovation is defined broadly as the development and implementation of new ideas, products, processes or organisational forms. Our use of the term in relation to health care encompasses service or quality improvement.*
   - *Follow up if necessary:*
     1. Was the innovation new or had it come from another setting?
     2. How did this influence adoption?
     3. If the innovation was adopted, what role did evidence play in this?
     4. How did evidence become a ‘tipping point’ for change?
2. **Preferences for evidence [20 mins]** *For the purposes of this exercise, evidence is defined broadly as* *including diverse forms of information, from academic research findings, local forms of data, e.g. audit reports, and stakeholders’ views.*
3. What types of evidence were used in your example of innovation or improvement?

*Prompts: research evidence (quant and qual), local data, expert opinion, patient need, implementation knowledge.*

*Do you think that the type of innovation influences the need for evidence or the type of evidence required/wanted?*

1. Where did the evidence come from?

*Prompts: Was it published in a peer-reviewed journal? Was it a national guideline? Who conducted the research/launched the guideline, and how reliable/independent were they?*

1. Who presented the evidence (i.e. who was the messenger/source and what effect did this have)?

*Prompts: Senior or junior member of staff and how did this influence its use? How much air time did it get and how did this influence its use? Internal/external person/body and how did this influence its use? Overall influence of credibility, personality and perceived expertise.*

1. (If multiple forms of evidence have been mentioned) How were the different types of evidence prioritised?
2. What other forms of evidence or information do you think should be used in decision-making?

*Prompts: How important is it to have certain information such as the number of deaths prevented/quality of life/costs/patient satisfaction/impact on inequalities etc. How does the evidence used align with your prior expectations of what would/should be used? Does this matter?*

1. What are the barriers to use of these other forms of information?

*Prompts: What might better enable their use in future? What determines whether or not a piece of evidence is used?*

1. Are there any other types of information that may not be framed as evidence but can/should be used as evidence?

**Patient specific questions**

- What constitutes evidence from patients?
- How is evidence concerning patients acquired?
- In what forms can evidence from patients be presented? Can you give some examples of what forms tend to be more effective? What impact does this have?
- How are these types of evidence received by different decision makers in different innovation contexts?
- How much of an impact does it have on the decisions that are made?
- What prevents it from having more of an impact?
- How does/can evidence from patients compete with other forms of evidence?
- Have there been any changes over time in the ways in which evidence from patients is used?

1. **Influence of professional processes on evidence use [20 mins]** *For the purposes of this exercise, professional processes are defined as the* *characteristics, standards, behaviour, values and guiding principles of different professionals (at the individual and group level) that may contribute in some way to use of evidence in decision making about innovation.*

a. B’GROUND. In your example of innovation, how do you think your professional background influenced your use of evidence?

- *Receptive to particular types of evidence (academic research, local data, knowledge gained through experience, patient perspective)*
- *Barriers too (e.g. economic evaluation, qual research, systematic reviews)*
- *Influence what evidence is presented (types of evidence); how it is interpreted (what picked out or emphasised); and applied (degree to which you are able to influence decision-making using evidence)?*
- *Plausibility to self versus others trying to influence?*

b. OTHER GROUPS. In your examples of innovations, how did other groups respond to the evidence?

- *doctors in acute or primary care*
- *doctors vs nurses and other allied health professionals*
- *managers vs clinicians*

c. TENSIONS. In your example of innovation, how were potential tensions between groups’ preferences resolved?

- *doctors and nursing/ allied health professionals – marginalize?*
- *managers’ and clinicians’ views – potential tensions?*
- *Commissioners versus service providers*
- *How does evidence of patient views fit in with professionals’ views?[differences across groups; credibility/perceived expertise]*

1. **Influence of organisational processes on evidence use [20 mins] DANIELLE** *For the purposes of this exercise, organisational processes are defined as* *organisational level values, beliefs, policies, structure and culture that may contribute in some way to use of evidence in decision making about innovation.*
   - In your example of innovation, what was the role of your organisation in evidence use and decision making?
   - How can your organisation either support or inhibit you in using evidence to make decisions about innovation?
   - What are the organisational level enablers and barriers to use of evidence in decisions about innovation?
     1. *Possible enablers:*

- *Well-informed organization (high level of peer awareness)*
- *Supportive infrastructure*
- *Leadership/managerial approaches (promoting evidence use)*
- *Culture (teamwork/institutional fashions)*
- *Data driven*
- *Promoting stakeholder involvement at all levels*
  - 1. *Possible barriers:*
- *Lack of time, resources and pressure*
- *Not receptive to innovation*
- *Lack of authority*
- *Degree to which evidence on innovation aligned with organisational needs*
- *Overall maturity/capability of the organisation to effectively use evidence to make decisions. For example, do they have the appropriate expertise (i.e. analysts), access to sufficient evidence, awareness of that access and opportunity to make use of it?*
  - How did these barriers/enablers influence the decision making around your innovation specifically? (This might already be covered as people respond to the first question)

1. **Influence of local system processes on evidence use [20 mins]** *For the purposes of this exercise, local system processes are defined as factors associated with the local health care system (i.e. beyond the limits and control of individual organisations and the individuals/groups within it) that may contribute in some way to use of evidence in decision making about innovation.*
   - ROLE In your example of innovation, were other organisations at the local system level relevant in decision-making or evidence use (e.g. commissioners, other providers, patient groups, AHSNs, clinical networks)?

- If so, how did they influence the use of evidence?
- Did they help to produce consensus around the adoption of innovation or was this challenging? What role did evidence play in this?
  - BARRIERS/ENABLERS In your example of innovation, were there barriers and enablers to use of evidence in decision-making at the local system level?
    1. *Possible enablers:*
- *legitimize need for innovation;*
- *enable wider range of stakeholder views (how negotiated?);*
- *influence behaviour at lower levels (e.g. by presenting evidence in particular ways to appeal)*
  - 1. *Possible barriers:*
- *system pressures, including austerity (‘what works’ over rigour, and preference for narrow economic information);*
- *not embedded enough in decision making at lower levels.*

1. **Are there any other factors/processes that we have not yet discussed that you think influence the use of evidence in decision making about innovation?**
